# Editorial: Global Control and Eradication Programmes for Cattle Diseases

**DOI:** 10.3389/fvets.2021.806111

**Published:** 2021-12-03

**Authors:** Beate Conrady, Nicola Decaro, David Graham, Julia Francis Ridpath, Inge Santman-Berends, Sam Strain, Jörn Gethmann, Matthias Schweizer

**Affiliations:** ^1^Department of Veterinary and Animal Sciences, Faculty of Health and Medical Sciences, University of Copenhagen, Copenhagen, Denmark; ^2^Complexity Science Hub Vienna, Vienna, Austria; ^3^Department of Veterinary Medicine, University of Bari Aldo Moro, Valenzano, Italy; ^4^Animal Health Ireland, Leitrim, Ireland; ^5^Ridpath Consulting, LLC, Gilbert, IA, United States; ^6^Department of Population Health Sciences, Faculty of Veterinary Medicine, Utrecht University, Utrecht, Netherlands; ^7^Royal GD, Deventer, Netherlands; ^8^Animal Health and Welfare Northern Ireland, Dungannon, United Kingdom; ^9^Friedrich-Loeffler-Institute, Federal Research Institute for Animal Health, Institute of Epidemiology, Greifswald, Germany; ^10^Institute of Virology and Immunology, Bern, Switzerland; ^11^Department of Infectious Diseases and Pathobiology, Vetsuisse Faculty, University of Bern, Bern, Switzerland

**Keywords:** animal health law, bacterial infection, bovine viral diarrhea virus (BVDV), cattle diseases, eradication, infectious bovine rhinotracheitis (IBR), mitigation programmes, viral infection

The disease status for a range of cattle diseases differs between countries and even between regions within countries. In Europe, several countries have implemented national or regional surveillance, control, or eradication programmes for infectious diseases that are not mandatory regulated in a harmonized way by the European Union (EU). Such diseases are listed under category C, D, or E in the New Animal Health Law (1) or are not listed at all (e.g., Bovine Viral Diarrhea (BVD), Infectious Bovine Rhinotracheitis (IBR), Salmonellosis, Paratuberculosis Johne's disease (JD), Enzootic Bovine Leukosis (EBL), Q-fever, Trichomonosis). Disease control programmes bring tangible benefits including improved animal health and welfare, reduced antibiotic use, and reduced direct and indirect farm production losses (2–5). Therefore, development and participation of farmers in disease control programmes at regional or national level are to be strongly recommended.

Trade has the potential to introduce infectious agents into regions where disease freedom has been achieved or where control measures have resulted in low disease prevalence. While there is a good overview and description of regulated diseases at the European level and control programmes are published on European websites, there is a lack of control programmes for cattle diseases that are not mandatory regulated by European regulations. Further, information on prevalence and control of such diseases outside Europe is fragmentary. Approaches and procedure of these control programmes, such as herd management, screening, surveillance or use of vaccines, vary widely between, and within countries. Knowledge of the experience gained in existing control programmes provides invaluable help to continuously improve them, and they can also serve as background knowledge for regions or countries that plan the design of new programmes. The aim of this Research Topic was to improve the knowledge of control and eradication programmes for cattle diseases in Europe and beyond with a special focus on “cattle diseases subject to no or limited mandatory regulation.” In total, 29 papers from 37 countries were published in this Research Topic, covering 31 different cattle diseases. Some of the papers in this special issue were submitted by researchers who participated in a European Cooperation in Science and Technology (COST) Action named SOUND-control, which runs from 2018 to 2022 [www.sound-control.eu (6)]. SOUND-control focuses on the topic of output-based surveillance for cattle diseases with either no or limited regulation under EU legislation. Limited regulation means that EU countries are not required to control the disease in their country. This COST Action provides an overview of national and regional control programmes for several cattle diseases. The contents of the New Animal Health Law [(EU) 2016/429 (1)] became available subsequent to the COST Action commencing. Therefore, many diseases formerly not included in the regulation are now listed as category C, D, or E in the New Animal Health Law, set into force in April 2021. For category C diseases, “measures are needed to prevent the disease from spreading to parts of the Union that are officially disease-free or that have eradication programmes” [2018/1882 (7)]. For category D diseases, “measures are needed to prevent the disease from spreading on account of their entry into the Union or movements between member states.” For category E diseases, surveillance within the Union is required (7). In practice, this means that there are no mandatory requirements to eradicate diseases listed as category C, D or E, nor input-based standards to demonstrate that a country is free from infections. However, in some situations, countries can set additional trade requirements depending on their national disease status for specific cattle diseases. Because of the absence of international standards, member states have either developed their own specific control programme or have no control programme at all. An output-based evaluation of these country-specific control programmes may support the validity of programme design and thus safe trade within Europe. Therefore, cattle diseases listed as category C, D, and E are included in SOUND-control and also within this Research Topic, as it remains relevant to obtain an overview of the existence and design of control programmes for cattle diseases across Europe. We refer to these diseases as diseases with “no mandatory regulation.” Although it was not the aim of the COST Action to include diseases listed in category A or B, some partners reported them. We have not excluded them from this Research Topic.

BVD and IBR with 20 and 10% of all papers, respectively, were the most discussed diseases in this special issue. The studies can be grouped into the following broad research areas: (i) overview of country specific control and eradication programmes and prevalence of cattle diseases (*n* = 22), (ii) development of online data tools to collect and/or assess epidemiological data for cattle diseases (*n* = 3), (iii) systematic reviews of risk factors for the disease introduction to cattle herds (*n* = 1), and (iv) effectiveness of different sampling materials to detect infected animals and immune responses of animals after vaccinations (*n* = 3).

The most comprehensive overview was provided by Hodnik, Acinger-Rogić et al. covering cattle diseases listed under categories C, D, or E in the Animal Health Law for which control programmes are in place within Europe. In this context, a survey in 33 countries was performed regarding country-specific control programmes for 23 diseases. The results show that the median number of control programmes implemented per country was six, ranging from one (Albania, Greece, and Macedonia) to 13 in Denmark. Overall, Norway had with 12 diseases the highest number of officially or perceived free statuses. EBL was the most frequently controlled disease (31 countries), whereby 22 countries were officially or perceived to be free. Approximately 86% of the programmes are implemented at national level, 75% covering both dairy and non-dairy cattle and 33% are implemented as voluntary programmes. Waldeck et al. contributed with a systematic literature review regarding risk factors for the introduction of bovine herpes virus 1 (BoHV-1) into cattle herds at EU level. The review covered 12 studies and showed that herd size, purchase of cattle, cattle density, age of cattle, distance to neighboring cattle herds and professional visitors were the most relevant factors for entrance of BoHV-1 into cattle herds.

Within the SOUND-control Cost Action, an online data collection tool was developed to evaluate data availability and quality and to collect outputs of different control programmes. The developed tool includes demographics of the cattle sector, risk factors for disease introduction, disease control programmes, and diagnostic strategies. BVD was used as the initial case disease for which the tool was developed. The authors describe the key learnings during the development of the tool (van Roon et al.). The study concludes that data requirements for different diseases can be generalized and readily addressed. Nonetheless, a high variability regarding data availability and comparability across European countries represents a challenge for integrating such data into standardized tools to assess freedom from infection in cattle herds (van Roon et al.). The online data collection tool was subsequently applied to assess the existence and quality of data and was evaluated for JD, IBR and BVD in 24 countries (Rapaliute et al.). The quality and quantitative availability of data on cattle demographics were better and more frequently available (70%) compared to risk factors (24%). Data related to control programmes for BVD were most commonly available (72%), followed by IBR (66%) and JD (34%).

Roch and Conrady provided an overview of prevalence, control and eradication programmes for EBL, IBR/infectious pustular vulvovaginitis (IPV), BVD, and bluetongue disease (BT) in Austria. The authors retraced regulations over a period of 42 years (1978–2020) to analyse the changes of legislation, focusing on sampling, testing and control activities, which were then linked to the diagnostic testing results of sampled animals. In this context, the modification of the legislation for these four cattle diseases related to their epidemiological situations over time was illustrated. Another study provides an overview of the control of 10 cattle diseases that were never detected in, or eradicated from, Finland and the control of a further 13 endemic or sporadic diseases (Autio et al.). For instance, <0.5% of the cattle herds are infected with Salmonella per year and 2% of the cattle herds were classified as infected with *Streptococcus agalactiae* at the end of 2020 (Autio et al.).

Nielsen et al. described the differences in purpose, principles, and design of control activities for JD, BVD and *Salmonella enterica* serotype Dublin in the Danish cattle population (Nielsen et al.). In contrast to the JD programme, the mitigation activities against *S. enterica* Dublin are mandatory, while BVD switched from an active control programme to a surveillance programme after successful eradication of the virus (BVDV) in 2006. Another overview study by Koleci and colleagues describes the available information and gaps in cattle disease control in Albania (Koleci et al.). In summary, most control activities exist for zoonotic diseases such as bovine brucellosis, bovine tuberculosis, and anthrax but no national mandatory control programmes are available for cattle diseases without mandatory regulation, only voluntary activities for IBR and BVD at regional level. Another study by Hodnik, Knific et al. focused on two voluntary control programmes for endemic cattle diseases (IBR and BVD) and three compulsory programmes for EBL (free status), anthrax (sporadic) and BT (currently perceived free) in Slovenia. The voluntary programmes are based on increased biosecurity, testing and culling or vaccination. The control of EBL is based on serological testing of a number of cattle herds and inspection of carcasses at slaughter or necropsy, whereas anthrax and BT are mainly controlled by application of vaccines. The principles of the voluntary programme for the control and eradication of BVD from infected herds in Slovenia is described in the study by Toplak et al. In total, 25% of tested cattle herds (*n* = 348) were positive for antibodies to BVDV. A detailed overview from 1976 to 2020 regarding BoHV-1 infections and associated surveillance in Slovenia is provided by Hostnik et al. In total, 204,662 sera of cattle older than 24 months were tested and BoHV-1-positive cattle were detected in 1,287 (3.6%) of the tested cattle herds in 2006 (Hostnik et al.).

Mandelik et al. described 10 years of the voluntary IBR control programme in Slovakia (from 1996 to 2006), in which only limited numbers of farms participated before it changed to a mandatory programme for all cattle herds at the end of 2006. In total, 60% of the cattle herds were IBR free in Slovakia in 2020. Another study from Romania provided a data report of the EBL surveillance programme between 2017 and 2020 (Irimia et al.). The outbreak incidences were significantly higher in the Danube Delta area compared to mainland area but with an observed reduction in the number of outbreaks during the study period in both areas (Irimia et al.).

The purpose of the study by Luzzago and Decaro was to provide an overview of the genetic diversity of pestiviruses circulating in the Italian cattle population. All three pestivirus species associated with BVD, BVDV1 (pestivirus species A), BVDV2 (pestivirus species B) and HoBi-like viruses (pestivirus species H; HoBiPeV) have been detected in cattle herds with different frequency and geographical occurrence. For instance, BVDV-1b and 1e have a wide distribution nationally, with a high frequency of 69.5% compared to other sub genotypes BVDV-1a, 1d, 1h, and 1k. BVD and others such as IBR and *Streptococcus agalactiae* are regulated at regional level. Tamba et al. highlighted in their study that EBL is almost eradicated, bovine tuberculosis and brucellosis are only present in southern Italy, while BT is endemic throughout the country. Mainly BTV-1, BTV-3, and BTV-4 are present in Italy (Tamba et al.). A further study described that four subgenotypes (BVDV-1a, 1b, 1c, and 2a) are circulating in the cattle population in Mexico (Gomez-Romero et al.). In general, the information for Mexico is limited because there is no requirement to notify BVD cases to the authorities and only voluntary control activities are implemented. By conducting a review, Bauermann and Ridpath presented the epidemiology and control of pestiviruses in Brazil. At least five subgroups (a–e) of the Pestivirus H are present, with subgroup-a of HoBiPeV being identified to date only in Brazil. Thus, the authors conclude that based on the reduced genetic variability, the opportunity exists to control the virus by using a vaccine with a single HoBiPeV subtype. The study by Tajima presents control activities for JD, EBL and BVD as well as associated prevalence from 2000 to 2019 in Japan. While JD is a regulated disease in Japan with compulsory surveillance activities, BVD and EBL are non-regulated and control activities are based on voluntary trials at regional level including usage of BVD vaccine.

The study by Van Duijn et al. evaluates the efficacy of the Dutch BVD control programme by evaluating the testing results regarding BVDV and/or antibodies of all cows >1 year of age from non-BVDV-free herds that are introduced into herds. The study shows that testing of cattle and their offspring is beneficial for the management of the risk of BVDV introduction, indicated by the detection of 67 BVDV-positive animals in 44 cattle herds in 2019. Santman-Berends et al. described the control programmes for six endemic cattle diseases in the Netherlands between 2009 and 2019 (i.e., BVD, IBR, Salmonellosis, JD, Leptospirosis, and Neosporosis). In the first step, the within-herd prevalence of infections is estimated when a herd enrolls in a control programme. In the second step, where infection is found, the herd enrolls in the control phase with elimination activities of the infection with a subsequent surveillance phase to monitor the free or low prevalence status. Most control programmes are tailored to dairy herds and participation for non-dairy herds is voluntary. The progress of the different control programmes in reducing the nationwide prevalence of the six diseases is monitored through regular prevalence surveys of which the results are presented in the paper as well.

For Northern Ireland, control programmes are available for BVD, JD, IBR, Leptospirosis and Neosporosis (Strain et al.). For more than 97% of all cattle alive at the end of 2020, a BVD test status could be assigned. The annual incidence of BVDV positive calves has decreased by 56% since 2016, mainly due to voluntary culling of PI animals by herd owners and a voluntary ban to slaughter BVDV positive animals in abattoirs to avoid rearing PI animals to reach the dead weight. A BVD Dashboard is available to graphically illustrate to Irish farmers the BVDV status of all animals currently in the herd, and also professional vet practices can use it to get an overview of the status of all herds to which they have been granted access (Guelbenzu-Gonzalo et al.). In addition to that, the authors described the associated data collection process in order to gain information about epidemiology including BVD prevalence and biosecurity practice of the farmers. In this context, the most widely identified plausible sources of infection included retained BVD-positive animals, trojan births (i.e., introduction into a herd of a pregnant animal unknowingly harboring a fetus persistently infected with BVDV), trade and indirect contact through farmers and other personnel in the absence of hygiene measures. Another study by Graham et al. described the organization, funding, challenges and progress of the Irish BVD programme. For instance, the prevalence of PI calves was reduced from 0.66% within 11.30% of cattle herds in 2013 to 0.03% within 0.55% of herds in 2020. The Irish JD control programme covers testing of the whole herd by ELISA on blood or milk samples and it is described in another study in this special issue (Gavey et al.). In total, 11% of the Irish dairy herds (n=1,750) were registered in the programme (as of end of December 2020) and more than 224,300 ELISA tests were conducted. The diagnostic results indicated that 8,466 (3.8%) ELISA tests were positive or inconclusive (Gavey et al.).

Schweizer et al. described the Swiss BVD mandatory eradication programme. In the first year of the programme, all animals were tested for evidence of being PI, followed by testing of all newborn calves for the next 4 years with antigen testing. Prevalence of calves being born PI decreased from 1.4% to <0.02%, followed by a change in the control activities to serological surveillance with a prohibition on vaccination. More than 99.5% of all cattle farms in Switzerland were free of BVDV, as of 2020. One of the main challenges described is to efficiently protect the cattle population from re-infection, e.g., due to the endemic presence of border disease virus (BDV) in the Swiss sheep population. It was estimated that approximately 10% of the Swiss cattle population are positive for antibodies to BDV. One benefit of BVD eradication in the Swiss cattle population is that BVD seroprevalence in sheep significantly decreased (Huser et al.).

The collection of the articles in this Research Topic nicely illustrates that a combined effort across borders is required to control these types of diseases, involving, e.g., basic and applied research and development, diagnostics, epidemiology, veterinarians, database management, legal authorities and last but not least, the farmers concerned. All the published papers in this Research Topic reflects the major efforts to improving the knowledge and filling gaps in the literature regarding control and eradication programmes for cattle diseases, in particular for cattle diseases without mandatory EU regulation i.e. categorized as C, D, E or not listed in the new Animal Health Law.

## Author Contributions

All authors drafted the editorial and contributed to the reviewing as well as editing the papers published in this Research Topic.

## Conflict of Interest

JR was employed by company Ridpath Consulting, LLC. The remaining authors declare that the research was conducted in the absence of any commercial or financial relationships that could be construed as a potential conflict of interest.

## Publisher's Note

All claims expressed in this article are solely those of the authors and do not necessarily represent those of their affiliated organizations, or those of the publisher, the editors and the reviewers. Any product that may be evaluated in this article, or claim that may be made by its manufacturer, is not guaranteed or endorsed by the publisher.
